# Epidemiology of osteoporotic fracture in Moldova and development of a country-specific FRAX model

**DOI:** 10.1007/s11657-019-0669-z

**Published:** 2020-01-28

**Authors:** Alla Zakroyeva, Olga Lesnyak, Victor Cazac, Liliana Groppa, Eugen Russu, Lia Chislari, Larisa Rotaru, Helena Johansson, Nicholas C. Harvey, Eugene McCloskey, Mattis Lorentzon, John A. Kanis

**Affiliations:** 1 Ural State Medical University, 3 Repina street, Yekaterinburg, Russia 620028; 2 North West State Medical University named after I.I. Mechnikov, 41, Kirochnaya street, St. Petersburg, Russia 191015; 3 State University of Medicine and Pharmacy, 165 Stefan cel Mare si Sfant blvd., 2004 Chisinau, Republic of Moldova; 4 Mary McKillop Health Institute, Catholic University of Australia, Melbourne, Australia; 5 MRC Lifecourse Epidemiology Unit, University of Southampton, Southampton, UK; 6 Centre for Metabolic Bone Diseases, University of Sheffield, Sheffield, UK; 7 Geriatric Medicine, Department of Internal Medicine and Clinical Nutrition, Institute of Medicine, University of Gothenburg, Gothenburg, Sweden; 8 Region Västra Götaland, Geriatric Medicine Clinic, Sahlgrenska University Hospital, Mölndal, Sweden

**Keywords:** FRAX, Fracture probability, Epidemiology, Hip fracture

## Abstract

**Summary:**

Retrospective population-based survey in 2 regions of the Republic of Moldova determined the incidence of fractures at the hip, proximal humerus and distal forearm. The estimated number of such fractures nationwide for 2015 was 11,271 and is predicted to increase to 15,863 in 2050. The hip fracture rates were used to create a FRAX model to help guide decisions about treatment.

**Objective:**

This paper describes the epidemiology of osteoporotic fractures in Republic of Moldova that was used to develop the country-specific fracture prediction FRAX® tool.

**Methods:**

We carried out a retrospective population-based survey in 2 regions of the Republic of Moldova (Anenii Noi district and Orhei district) representing approximately 6% of the country’s population. We identified hip, forearm and humerus fractures in 2011 and 2012 from hospital registers and primary care sources. Age- and sex-specific incidence of hip fracture and national mortality rates were incorporated into a FRAX model for Moldova. Fracture probabilities were compared with those from neighbouring countries having FRAX models.

**Results:**

The incidence of hip fracture applied nationally suggested that the estimated number of hip fractures nationwide in persons over the age of 50 years for 2015 was 3911 and is predicted to increase by 60% to 6492 in 2050. Hip fracture incidence was a good predictor of forearm and humeral fractures. FRAX-based probabilities were higher in Moldova than neighbouring countries (Ukraine and Romania).

**Conclusion:**

The FRAX model should enhance accuracy of determining fracture probability among the Moldavan population and help guide decisions about treatment.

## Introduction

The demographic transition caused by the increase in life expectancy and change in lifestyle pose challenges to modern health care systems due to the social and health problems associated with aging. Among these challenges is the rising prevalence of osteoporosis worldwide, and the colossal medical and economic consequences of fragility fractures. In Europe, the annual cost of fractures associated with osteoporosis exceeded € 37 billion in 2010 [1] and disability due to fragility fracture was greater than that caused by any single cancer, with the exception of lung cancer. Disability was comparable or greater than that lost to a variety of chronic non-communicable diseases, such as rheumatoid arthritis–, asthma- and high blood pressure–related heart disease [2]. In women over 45 years of age, fragility fractures account for more days spent in hospital than many other diseases, including diabetes, myocardial infarction and breast cancer [2].

Fortunately, a wide variety of treatments is available that favourably affect bone mass and thereby decrease the risk of fractures associated with osteoporosis [3]. The use of such interventions by health care practitioners is assisted by instruments that assess patients’ fracture risk to optimise clinical decisions about prevention and treatment. The most widely used web-based tool FRAX® (https://www.sheffield.ac.uk/FRAX/) meets these requirements and computes the 10-year probability of low energy fractures based on several common clinical risk factors and, optionally, a DXA scan result [4, 5]. Specifically, FRAX models compute the probabilities of major osteoporotic and hip fracture derived from the risk of fracture and the competing risk of death, both of which vary from country to country. The development of country-specific FRAX models requires information on fracture incidence and death [4]. Until recently, no FRAX model was available for Moldova due to the lack of appropriate epidemiological data. This paper describes the acquisition of data for the creation of a country-specific FRAX model for the Republic of Moldova.

## Methods

The present study is a component part of the Multicenter Multinational population-based Study in Eurasian Countries (EVA study or ЭВА, in Russian). The broad aim of the study was to provide epidemiological information on fracture risk so that FRAX models could be created for Russia [6], Armenia [7], Belarus [8], Moldova, Kazakhstan and Uzbekistan. The present report describes the epidemiology of fractures at the hip, forearm and humerus in Moldova and the generation of a country-specific FRAX model.

The Republic of Moldova is a landlocked country in Eastern Europe bordered by Romania to the west and Ukraine to the north, east, and south. In 2010, the population of Moldova was 3,563,695 [9] but this excludes 520,786 people that lived in the breakaway state of Transnistria.

For the present study, we chose two areas of the country Anenii Noi and Orhei districts in central Moldova with a predominantly rural population. These districts were chosen for the ease of access to all medical records. The well-defined catchment areas ensured that the sources of medical record were comprehensive. The catchment population for the study period of 2011–2012 comprised 83,144 individuals from Anenii Noi and 125,866 from Orhei. Thus, the total catchment population of the two regions was 209,010 representing 5.1% of the total population (or 5.6% excluding Transnistria). Eighty percent of the catchment population were from rural communities which is higher than the national average. According to the 2014 census, the percentage of Moldovans living in rural areas was 62% [9]. The age and sex distribution was very similar to that of the whole country. The ethnic distribution was Moldovan (85.5%), Ukrainian (5.6%), Romanian (4.6%) and Russian (3.2%) similar to that recorded in the national census of 2004 (76%, 8.4%, 2.2%, and 5.9% for Moldovans, Ukrainians, Romanians and Russians, respectively [9]).

The retrospective population-based study covered a 24-month period from 1 January 2011 to 31 December 2012. In both locations, the medical records of all fractures in men and women aged 40 years or older were retrieved from the central city hospital registers (one hospital for each region), outpatient trauma units, and emergency services, 27 primary care centres and 2 private centres of medical care. The data on the following low energy fractures were collected: hip (ICD-10 codes S72.0, S72.1, S72.2), distal forearm (S52.5, S52.6) and proximal humerus fracture (S 42.2). Cases of high energy fractures were excluded from the analysis.

The reason for accessing multiple sources of information including that from primary care was to identify patients with hip fracture who were not admitted to hospital. The reason for this strategy was the observation that many patients in Eastern Europe are not hospitalized because facilities for surgical management are limited so that hospital admission is not feasible. In Belarus, for example, 29% cases of hip fracture did not come to hospital attention [8]. High rates of non-admittance have been reported in Armenia (44%) [7], Pervouralsk in Russia (27%) [6], Georgia (75%), Kazakhstan (50%) and Kyrgyzstan (50%) [10]. These missing cases from hospital discharge data reinforce a view that data on hip fracture based solely from hospital records are unreliable in this region of the world.

Only fractures validated by radiographs were included. To avoid double counting, further admissions for the same fracture site in the observation time were excluded. In some documents, fracture ICD-10 code was not specified. In such cases, radiographs were retrieved and verified fractures were included in the database. Permanent residence in the region was not a criterion for inclusion, so a small number of patients living temporarily in the catchment area (*n* = 33) were also included in the database. Yearly incidence rates were estimated from the number of men and women in 10-year age intervals with at least one index fracture in 2011 and 2012 divided by the age- and sex-specific population.

The age- and sex-specific incidence in 2011 and 2012 was applied to the Moldovan population for 2015 to estimate the number of hip, forearm and humeral fractures nationwide. Additionally, future projections were estimated up to 2050 assuming that the age- and sex-specific incidence remained stable. Population demography was taken from the United Nations using the medium variant for fertility [11].

The data on hip fracture were used to construct the FRAX model. For other major osteoporotic fractures (clinical spine, forearm and humeral fractures), it was assumed that the age- and sex-specific ratios of these fractures to hip fracture risk found in Sweden were comparable with those in Moldova. This assumption has been used for many of the FRAX models with incomplete epidemiological information. Available information suggests that the age- and sex-stratified pattern of fracture is very similar in the Western world and Australia [12–14]. In order to test this further, we compared the incidence of a forearm or humeral fracture observed in Moldova with the incidence that would be predicted from the pattern of incidence in Malmo applied to the incidence of hip fracture in Moldova. This assumes that the age- and sex-specific pattern of incidence of proximal humerus and forearm fracture (i.e. other major fractures; OMF) and hip fracture (HF) in Moldova is similar to that seen in Malmo [12]. Thus, for each age and sex,

$$ \frac{{\mathrm{HF}}_{\mathrm{Moldova}}}{{\mathrm{HF}}_{\mathrm{Malmo}}}=\frac{{\mathrm{OMF}}_{\mathrm{Moldova}}}{{\mathrm{OMF}}_{\mathrm{Malmo}}} $$

therefore,

$$ {\mathrm{OMF}}_{\mathrm{Moldova}}=\frac{{\mathrm{HF}}_{\mathrm{Moldova}}\times {\mathrm{OMF}}_{\mathrm{Malmo}}}{{\mathrm{HF}}_{\mathrm{Malmo}}} $$

From this, the incidence of a forearm or humerus fracture, estimated using the Malmo ratios, was compared with the empirical data from Moldova. The analysis was confined to women where the numbers of fractures were higher.

The development and validation of FRAX have been extensively described [4, 5]. The risk factors used were based on a systematic set of meta-analyses of population-based cohorts worldwide and validated in independent cohorts with over 1 million patient-years of follow-up. The construct of the FRAX model for Moldova retained the beta coefficients of the risk factors in the original FRAX model with the incidence rates of hip fracture and mortality rates for Moldova. National mortality rates used data from the United Nations for 2009 [15]. Ten-year fracture probabilities were compared to those of neighbouring countries (Romania and Ukraine).

In order to compare Moldovan hip fracture probabilities with those of other regions of the world, the remaining lifetime probability of hip fracture from the age of 50 years was calculated for men and women, as described by Kanis et al. [16]. In the present analysis, values for Moldova were compared with those of Bulgaria, China (Hong Kong), Canada, Denmark, Finland, France, Greece, Kazakhstan, Poland, Portugal, Romania, Russia, Spain, Sweden, Turkey, Ukraine, the UK and the USA.

## Results

In 2011–2012, a total of 1035 fractures were identified in individuals age 40 years or more. These comprised 340 hip fractures, 197 humerus and 494 distal forearm fractures.

### Hip fracture

A total of 137 hip fractures were identified in men and 203 in women (female/male ratio 1.5). Below the age of 70 years, hip fractures were more prevalent in men than in women (female/male ratio 0.8) but thereafter were more frequent in women (female/male ratio 3.3). The incidence of hip fracture increased with age in men and women, though more markedly in women (Table 1). Of the 340 cases with hip fractures, 334 were hospitalized.

**Table 1:** Population of the catchment areas, number of hip fractures and annual incidence of hip fractures (rate/100,000) in men and women in Moldova by age based on population data of from Anenii Noi and Orhei districts of Moldova for 2011 and 2012

Age (years)	Population	Hip fractures*	Incidence/100,000	95% CI
Men				
40–49	13,312	15	56.3	31.5–93.0
50–59	14,353	47	163.7	120.3–217.7
60–69	6827	36	263.7	184.6–365.1
70–79	3445	30	435.4	293.7–621.7
80–89	734	8	545.0	235.1–1074
90+	71	1	704.2	14.1–3929
40+	38,739	137	176,83	148.4–209.0
Women				
40–49	14,880	5	16.8	5.4–39.2
50–59	16,947	29	85.9	57.3–122.9
60–69	8921	41	229.8	164.9–311.8
70–79	5789	69	596.0	463.7–754.3
80–89	1982	48	1211	892.8–1606
90+	158	11	3481	1735–6230
40+	48,674	203	208,53	180.8–239.3

*Fractures over 2 years

### Forearm and humeral fractures

Fractures at the distal forearm were more frequent in women than in men (female/male ratio = 3.5). Fracture incidence in women rose with age up to the age of 69 years and thereafter decreased with age. In men, the incidence of forearm fractures decreased with age (Table 2). The annual incidence of proximal humerus fractures was lower in men than in women (female/male ratio = 2.8). Humeral fractures were less common than forearm fractures and the association with age less secure.

**Table 2:** Number and annual incidence of forearm and humeral fractures (rate/100,000) in men and women in Moldova by age based on population data of from Anenii Noi and Orhei districts of Moldova for 2011 and 2012

Age (years)	Forearm			Humerus		
	Fractures*	Incidence	95% CI	Fractures*	Incidence	95% CI
Men						
40–49	24	90	58–134	5	19	6–44
50–59	53	185	138–242	22	77	48–116
60–69	25	183	118–270	14	103	56–172
70–79	8	116	50–299	9	131	60–248
80–89	1	68	1–380	2	136	16–493
40+	111	144	118–173	52	67	50–88
Women						
40–49	35	118	82–164	7	24	9–48
50–59	151	446	377–523	49	145	107–191
60–69	120	673	558–804	39	219	155–299
70–79	61	527	403–677	39	337	239–461
80–89	16	404	231–656	11	277	138–497
40+	383	395	356–436	145	149	126–176

*Fractures over 2 years

### Fracture projections

Assuming that the fracture rates in Anenii Noi and Orhei districts were representative for the whole country, and based on the UN estimates of Moldavan population for 2015, we estimated that the annual number of all three types of fracture in men and women age 50 years and older in Moldova in 2015 was 11,271, comprising 3911 hip fractures, 5216 distal forearm fractures and 2144 humerus fractures. The number of fractures is expected to increase progressively over calendar year with an increase of 41% for the three fracture sites by 2050 (Table 3). The increase in hip fracture numbers is particularly great in women (81%) due to the high age dependency of hip fracture incidence.

**Table 3:** Estimated total number of hip forearm and humerus fractures in men and in women age 50 years and older in 2015 projected up to 2050 in Moldova

	2015	2020	2030	2040	2050
Hip fracture ^a^					
Men	1359	1375	1526	1644	1818
Women	2381	2617	3177	3813	4359
Forearm fracture^b^					
Men	914	875	903	1146	1127
Women	3789	3986	4258	4772	4953
Humerus fracture^c^					
Men	513	513	542	651	714
Women	1528	1571	1806	2041	2113
Totals	10,484	10,937	12,212	14,067	15,084

^a^Hip (ICD-10 codes S72.0, S72.1, S72.2)

^b^Distal forearm (ICD-10 codes S52.5, S52.6)

^c^Humerus (ICD-10 code S 42.2)

### Fracture probability

The incidence of forearm and humeral fractures was very similar to that predicted from the epidemiology of fracture in Malmo (Table 4) and the Malmo ratios were used for the construct of FRAX. Overall, the incidence of fractures at the distal forearm and humerus was well predicted by the use of the Malmo ratios to hip fracture incidence from Moldova. The exception in women for forearm fracture was at the ages of 55–59 years where the use of Malmo ratios overestimated the observed incidence. At other ages, the estimates lay within the 95% confidence intervals of the empirical data. For humerus fracture in women, the exception was between the ages of 60–64 years where the use of Malmo ratios underestimated the observed incidence.

**Table 4:** The incidence of forearm and humeral fractures in women predicted from the epidemiology in Malmo (see methods) and that observed in the present study with 95% confidence intervals (CI). The observation in bold denote a difference between observed and predicted estimates

Age (years)	Forearm			Humerus		
	Predicted	Observed	95% CI	Predicted	Observed	95% CI
50–54	447	412	324–516	133	99	59–156
55–59	671	**485**	**382–607**	188	198	134–281
60–64	590	705	558–879	132	**250**	**166–361**
65–69	539	617	443–838	274	166	83–296
70–74	606	560	397–769	257	324	203–491
75–79	407	479	304–720	247	354	206–568
80–84	468	407	203–729	220	296	128–584
85–89	278	397	128–925	227	238	49–695

**Fig. 1:**
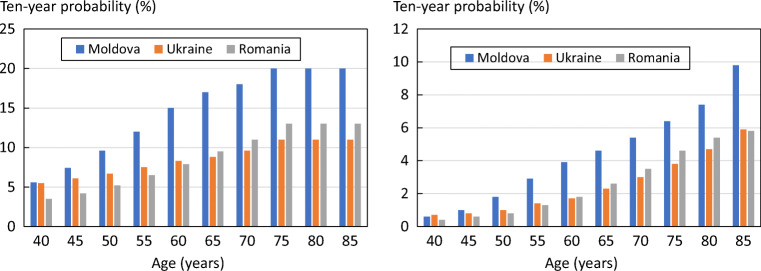
10-year probability of a major osteoporotic fracture (left hand panel) and hip fracture (right) in women with a prior fracture by age from Moldova, Ukraine and Romania. Body mass index set to 25 kg/m^2^

The 10-year probability of major osteoporotic fracture and hip fracture in Moldova and neighbouring countries is shown in Fig. 1 in women with a prior fracture by age. Ten-year probabilities were consistently higher than in the neighbouring countries of Ukraine and Romania.

Lifetime probabilities for hip fracture are shown in Table 5.

**Table 5:** Lifetime probability of hip fracture in the Moldovan population from the age of 50 years compared with selected countries. From [16] except where indicated

Country	Lifetime risk at 50 years %	
	Women	Men
Sweden	25.6	11.0
Denmark	23.0	11.3
France	19.3	5.9
China (Hong Kong)	17.7	7.6
USA (Caucasian)	16.1	7.5
Turkey ^a^	15.9	3.6
Canada	15.5	5.8
Greece	15.4	6.8
UK	14.4	5.0
Portugal	13.7	4.8
Finland	12.9	6.0
Kazakhstan	12.6	6.0
Spain	12.6	4.2
Bulgaria	11.2	4.4
Hungary	10.8	4.2
Mexico^b^	10.6	5.0
Poland ^f^	10.1	4.2
Moldova ^e^	9.3	5.7
Russia ^c^	7.7	3.8
Romaia ^d^	7.0	3.8
Ukraine ^g^	5.6	2.9

^a^ [17]; ^b^ [18]; ^c^ [6]; ^d^ [19]; ^e^ This study; ^f^ [20]; ^g^ [21]

## Discussion

This study documented the incidence of hip, distal forearm and proximal humeral fragility fractures in Moldova based on regional estimates from two districts. As expected, hip fractures were more frequent in women than in men (female/male ratio = 1.48). In both sexes, the incidence increased with age. It is of interest that for people younger than 70 years, the hip fracture rate among men was higher than in women. Thereafter, incidence was higher in women. Similar results have been reported in many studies including other countries of the EVA project, namely Russia, Armenia and Belarus [6–9]. From these results, Moldova belongs to the moderate-risk countries for osteoporotic hip fracture for men and women [22].

Based on the regional incidence, the number of hip fractures in 2015 was estimated at 3911 and is expected to increase by 65% to 6492 in 2050. These estimates are relatively robust in that all individuals who will be aged 60 years or more in 2050 are currently adults. However, these estimates may be conservative since they assume that the age- and sex-specific risk of hip fracture remains unchanged over this period. Decreases in age-specific rates have occurred in those countries with the higher hip fracture risks [23], whereas increases in incidence with time are commonly found in those countries with the lower risks. It is estimated that modest increases in secular trends (e.g. 1% per year) as seen for example in Mexico [24] together with demographic changes would double the number of hip fractures over 20 years [25]. For hip, humerus and forearm fractures combined, the numbers anticipated will increase by 41%. Such projections are important for health care planning.

Ten-year probabilities were consistently higher than in the neighbouring countries of Ukraine and Romania. These differences in fracture probability cannot be accounted for by differences in mortality but rather, reflect differences in the risk of hip fracture. Reasons for the heterogeneity in hip fracture risk are speculative [24]. The factor which best predicts the heterogeneity in hip fracture risk is socioeconomic prosperity that in turn may be related to low levels of physical activity [26]. The fact that there are differences in adjacent countries emphasizes the importance of the use of country-specific FRAX models rather than surrogate models [27].

A minority of countries that have a FRAX model also have robust information on the risk of other major osteoporotic fractures. In the absence of such information, FRAX models are based on the assumption that the age- and sex-specific pattern of these fractures is similar to that observed in Malmo [28]. The acquisition of data on the incidence of forearm and humerus fractures in a manner identical to that for hip fracture permitted the adequacy of this assumption to be tested, at least for forearm and humeral fractures. Our findings suggest that the incidence of forearm and humerus fractures can be reasonably predicted from the incidence of hip fracture. Very similar findings have been reported from Canada [14], Iceland [13], the USA [29], the UK [30], Australia [31] and several additional counties of the Western world, despite differences in incidence [28, 32]. This commonality of pattern is supported by register studies, which indicate that in those regions where hip fracture rates are high, so too is the risk of forearm fracture and spine fractures (requiring hospital admission) [33, 34]. To our knowledge, the present study is the first to report the commonality of fracture pattern in Eastern Europe.

There are a number of limitations to this study. With regard to fracture incidence, we examined only about 5% of the Moldovan population. Therefore, the extrapolation of these regional estimations to the entire country is an assumption that we were unable to test. In addition to large variations in fracture rates around the world, fracture rates may vary within countries. In addition to ethnic-specific differences [35], up to two-fold differences in hip fracture incidence have been reported using common methodology with the higher rates in urban communities including Croatia [36], Switzerland [37], Norway [38], Argentina [39] and Turkey [40].

Despite the rigour of the methodology and well-defined catchment population, it is possible that not all hip fractures were captured. It is relevant, however, that accuracy errors have little impact on the rank order with which the FRAX tool categorizes risk in a given population [7, 41] but they do change the absolute number generated and thus have implications where treatment guidelines are based on cost-effectiveness or the economic burden of disease. In order to address these limitations, representative populations representative of the general population at risk would need to be studied prospectively, preferably over a 10-year time horizon.

In summary, a FRAX model has been created for the Republic of Moldova that based on a regional population-based estimates of the incidence of low energy hip fractures. The model should enhance accuracy of determining fracture probability among the Moldavan population and help to guide decisions about treatment.
